# Measuring data access and re-use in the European Legal Framework for Data, from the General Data Protection Regulation (GDPR) law to the Proposed Data Act: the case of vehicle data

**DOI:** 10.12688/openreseurope.16468.1

**Published:** 2023-11-07

**Authors:** Tommaso Crepax, Mitisha Gaur, Barbara da Rosa Lazarotto

**Affiliations:** 1 Dirpolis, Sant'Anna School of Advanced Studies, Pisa, Pisa, Italy; 2 Law Science Technology and Society, Vrije Universiteit Brussel, Brussels, Brussels, Belgium

**Keywords:** Data Portability, Data Reuse, Data Governance, Event Data Recorder, Access to Vehicle Data

## Abstract

This article delves into the difficulties and opportunities associated with the acquisition, sharing, and re-purposing of vehicle data, particularly information derived from black boxes used by insurance companies and event data recorders installed by manufacturers. While this data is usually utilized by insurers and car makers, it may benefit consumers, rival firms, and public institutions profiting from access to data for objectives such as data portability between insurance companies, traffic and transportation management, and the development of intelligent mobility solutions. Among other regulations, the authors examine the proposed Data Act as the European chosen champion to address the legal and technical hurdles surrounding the reuse of privately held corporate data, including privacy and intellectual property, competition, and data interoperability issues. The text also offers an overview of the sorts of data obtained through vehicle recording systems and their potential benefits for various stakeholders. This paper presents a methodology for comparing and evaluating, in an ordinal fashion, the degree of access conferred by various regulations and put it to practical use to assess how much data is currently left out from access by the existing legislation, how much of such data is covered by the Data Act, and ultimately, how much still remains inaccessible for reuse.

## Plain language summary

This research paper explores the challenges and possibilities related to gathering, sharing, and reusing data from vehicles, specifically data from black boxes used by insurance companies and event data recorders installed by manufacturers. While this data is usually used by insurers and car makers, it could also benefit consumers, competing firms, and public institutions. For example, it could help with transferring data between insurance companies, managing traffic and transportation, and developing smart mobility solutions. The authors also discuss the proposed Data Act, which is a European regulation aimed at addressing legal and technical obstacles related to reusing privately held corporate data. This involves issues like privacy, intellectual property, competition, and data compatibility problems. The paper provides an overview of the different types of data obtained through vehicle recording systems and their potential advantages for various stakeholders. Furthermore, the paper introduces a method for comparing and evaluating different regulations to determine how much data is currently inaccessible under existing laws, how much is covered by the Data Act, and how much data is still unavailable for reuse.

## 1   Introduction

It is a common industry practice that insurance companies collect people’s driving and vehicle usage data in cars’ black boxes to better tailor their insurance packages and services ^1^, as well as evaluate and manage their own financial risks ^2^. Additionally, recent international (UN) ^3^ and European regulations ^4^ require car manufacturers to install Event Data Recorders (’EDR’) to improve safety. In both cases, the more accurate the data, the better its quality, and the better the business decision-making based on the information mined from the car’s ’sensing systems’.

These qualitatively sound datasets so created are normally used for the commercial benefit of either the insurance company or car manufacturer alone ^5^ and have no envisaged secondary use for the individual, the market, or the public ^5^. However, as is also highlighted by the European Commission in the Access to Vehicle Data initiative, ^1^ all such parties are potential stakeholders of the rich datasets and have multiple interests in their access and re-use. The after-sales economic potential in the re-use of such data is substantial, both in economic and social terms ^7^. Consumers might want to port data demonstrating their good driving habits to another insurance company and get better offers for insurance premiums and policy coverage. On the other hand, approaching the situation from a commercial perspective competing insurance companies could through using these datasets avoid costly processes such as data collection, analysis, and profiling of new customers. Additionally, public bodies such as municipalities could use these privately held datasets to perform public-purpose tasks, such as traffic and public transportation management, but also combine them with other available datasets and develop new smart mobility solutions for neighbouring public purposes, such as combating pollution.

Meanwhile, the development of the Internet of Things (IoT) systems and market uptake of connected cars keeps changing the paradigm from quasi-ownership of data by insurance companies and data holdership by cars manufacturers, to also Internet Service Providers (ISPs), software developers, along with changes in the power structures of the market and brokerage for such data.

It is evident how the digital economy is evolving along with new technologies, but its need for quality data has remained unanswered. In fact, access and re-use of privately held data are hindered by legal issues of privacy, data protection, intellectual property, competition, and technical issues of data such as data formats as well as services interoperability.

Nowadays, challenges related to data access are being tackled by legal instruments such as the EU General Data Protection Regulation (GDPR) and the Database Directive. These instruments define the rights of data subjects regarding their personal data and provide exclusive ownership rights to database producers, yet simultaneously facilitate and restrict access to data depending on relational circumstances ^7, p. 20^.

To the same challenges, the proposed Data Act puts forward solutions ranging from clarifying rules on data sharing, individuating venues for defining portability and interoperability of data and services and creating new access rights. As for the latter, the interest is in the implications of the newly formulated specification of the Business-to-Government (B2G) data-sharing exception (recital 58 of the second presidency compromise text) where a public sector body can ask for access to privately held data is in charge of a specific task in the public interest related to local transportation or urban planning, enhancing infrastructure services, or generating accurate and current statistical data.

This research paper focuses on examining the feasibility of the Data Act in relation to the access and portability of consumers’ personal driving data. Specifically, it analyzes the legal and technical obstacles presented by the proposed legislation, with a particular emphasis on the potential hindrances to the complete re-usability of privately held business data.

## 2   Methods

### 2.1   Research objectives

The primary goal of this study is to assess the effectiveness of the current legal framework in the European Union regarding data access and reuse, in comparison to what is envisioned in the data strategy. Specifically, the study focuses on access and reuse of data from insurance-owned blackboxes and manufacturers’ owned event data recorders.

### 2.2   Structure of the paper

To achieve this objective, Section 2 focuses on the indepth analysis of the process of identifying, analyzing, and categorizing vehicle data records, clustered into ’data elements’ (driver data, vehicle data, and else data as defined in Section 3.1). Section 3 identifies the three primary stakeholders, namely individual users or consumers of digital services, businesses, and public sector bodies, interested in accessing and reusing data over which they have no practical control. ^2^ Section 4 then examines the relevant articles in the GDPR, FFNPD Regulation, and Open Data Directive to identify provisions that establish rights, or provide access and reuse opportunities for each stakeholder. This initial step enables to reach the first Milestone that is, to determine the extent to which data recorded in black boxes or event data recorders, can be accessed through existing legal mechanisms. Furthermore, in order to obtain a more precise measurement of the access, the latter is subdivided into the four fundamental operations of Create, Read, Update, and Delete (CRUD) that are commonly used in relational database management systems (RDBMS) and other software systems that involve data storage and manipulation. ^3^ The CRUD operations are a versatile classification system encompassing a broad range of actions that can be performed on a dataset, however they leave out a few rights, such as that of the individual to request the controller for its data to be downloaded, or sent directly to (as in Art. 20 GDPR) another controller, or for the consumer to have any data made accessible by its holder (Art. 3 of the Proposed Data Act).

It can be reasonably assumed that having only the ability to read a specific dataset leads to a lower level of access or control, as compared to the additional capabilities of creating, updating, deleting, downloading, and so forth, which further enhance the level of access. The same holds true for the degree of accessibility, in regards to either some data or all, as well as to first-party and third-party access. Therefore, utilizing this framework, the evaluation of the extent of control can be achieved not much through numerical values, but via comparative analysis, which is referred to as ’ordinal’ categorization (greater than, smaller than, equal to, etc.). In other words, it enables the evaluation of whether a new legal text confers greater or lesser ’access’ authority to a stakeholder.

Section 5 then analyzes the Data Act to ascertain the extent to which it further extends the scope of data access and use per stakeholder for each Data Element, which constitutes the second Milestone. Section 6 covers promising research areas that have not been extensively discussed. These areas include topics such as competition, data governance and markets, and quality of data. Finally, Section 7 concludes the paper, summarizing the key findings and providing suggestions for future research directions.

In this paper we have developed a standardized schema to determine which stakeholder has access to what data and for what specific purpose, available as a separate resource ^8, 9^.The schema helps to streamline and enhance the understanding of access regimes while facilitating the creation of a machine-readable version for easier automation or embedding into software. By adopting this schema, the authors aim to provide a practical solution for enhancing data access and reuse while improving transparency and accountability in the process.

In summary, this study adopts a systematic approach to evaluate the extent to which the existing EU legal framework on data delivers on the promise of access and reuse of data as outlined in the data strategy. The comprehensive methodology involves identifying stakeholders, examining relevant data regulations, analyzing the data act, evaluating remaining data, and creating a standardized schema for enhanced understanding and automation. The study provides valuable insights and recommendations for enhancing data access and reuse while improving transparency and accountability in the process.

## 3   Datasets of vehicle data

This section of the paper focuses on the process of individuating, analyzing, and classifying vehicles’ data collection mechanisms, which are datasets comprising information captured by the recording systems integrated into the vehicle.

This analysis includes an examination of data obtained from two types of recording systems: insurance-based black boxes (iBBs) and manufacturer-installed event data recorders (mEDRs). While these systems are similar in terms of their technological aspects, the legal and economic implications associated with them are quite distinct.

In terms of the individuation of data collected by recording systems, Annex 4 of the "UN Regulation No. 160: Uniform provisions concerning the approval of motor vehicles with regard to the Event Data Recorder" ("EDR Regulation") presents a comprehensive inventory of the required data to be recorded by the system in the event of specific incidents. The list of data is quite detailed, and each individual data point has the potential to yield valuable insights for various purposes and stakeholders.

### 3.1   Clusters of data elements

In the EDR Regulation ^3^, Annex 4 lists 41 data elements that the recorder collects when the specific event happens (’triggering event’). Data elements range from the indicated speed of the vehicle, the percentage of engine throttle, the status of the driver’s safety belt (not/fastened), the time to deploy the frontal air bag, the normal, lateral and latitudinal accelerations, to unusual accelerations in any direction, the anti-lock brake system (ABS) activity, the status of the stability control, through the seat track position of the driver, the front or driver passenger occupant size classification (adult, female, child) and so on. The reason for listing so many of the data elements is to show that each element can be used, alone or jointly, to prove, infer and support information about the situation around a driving episode. Noteworthy information about such situations could relate to individuals in the car, such as that there was a child in it or that the driver was presumably drunk, or relate to the car itself, such as that the car malfunctioned at the time of the accident, was hit from this and not that angle or it was first hit in the front and then in the back.

While it would certainly be of research interest to link each data element to a particular use case for stakeholders, to maintain brevity and stay within the scope of this paper, it opted to group the data into three main categories: data that pertain to the driver (’driver data’), data that pertain to the vehicle systems (’vehicle data’), and data that pertain to external factors, such as other vehicles, people, environment, traffic, and so on (’else data’). The data elements have been grouped into the aforementioned categories in accordance with rules to ensure that the categories are comprehensive and encompass not only data generated by driver actions or by the vehicle independently, but also data that can provide insights about the driver, the vehicle, or external factors (such as the driver’s state of mind inferred from accelerations or sudden brakes). It is important to note that a single data element can belong to multiple categories. For instance, it is possible to draw inferences about the vehicle’s status from the driver’s behaviours (such as constant braking due to strange acceleration), and also draw inferences about the driver’s state from the vehicle’s functioning (such as frequent lane corrections).

### 3.2   Black boxes

As of July 2022, all cars produced in the European Union are required to be equipped with an event data recorder (mEDR). The technical specifications of these devices are governed by UN Regulation No. 160, ^4^ which sets forth the types of data to be recorded by the mEDR and the events that trigger solid-state recordings. In addition, there are regulations and communications at the EU level, including Directive 85/374/CEE, Regulation (EU) 2019/2144, and the C-ITS Communication, which establishes requirements for motor vehicle safety and the protection of vehicle occupants and vulnerable road users. ^5^

Until now, the practice has seen some car insurers propose– and in some cases require—their users to install insurance black boxes to facilitate a personalized insurance premium. The installation of iBBs in cars entails that data is collected by insurance companies, and it is important to understand the legal and privacy implications of such data collection.

An iBB refers to a data recorder that insurance companies install in customers’ cars. The data collected by the iBB is under the possession of the insurance company (the *de facto* holder). The legal basis for the installation of iBBs is contractual, and the purpose of their installation is in the interest of the insurance company.

Although the iBB can potentially collect a vast array of data, privacy regulations such as the US Driver Privacy Act, general data governance regulations such as the General Data Protection Regulation (GDPR), special regulations such as the ePrivacy Directive or Database Directive, other EU vertical regulations specific to vehicles and digital systems, ^6^ and more, restrict the scope of data that can be collected, accessed, processed, and shared. In addition to, and complementing legal obligations and duties, there are security and product requirements playing a role in such access landscape. For instance, to comply with data minimization principles, iBBs must implement technical controls to allow for storage of only relevant event data in solid-state memory, while the rest of the non-relevant data is stored and overwritten in volatile memory.

This complex system of legal and practical access causes that, although insurance companies have material possession of the iBB datasets, their access to data is only granted for legitimate reasons. Consumers, on the other hand, who are not in practical possession of the iBB data, can exert rights over them, although in a limited fashion and only to a certain extent.

In summary, the use of iBBs for car insurance purposes presents a balancing act between the potential benefits of lower premiums for consumers and the potential loss of autonomy. It is important to adhere to data protection principles while ensuring that the legitimate interests of both parties are maintained.

### 3.3   Event data recorder

There are several differences between iBBs and mEDRs that are relevant when examining the data collected by these devices.

The first one is that mEDRs are installed directly by car manufacturers, and the data collected by these devices are held by them, while in contrast, iBBs are installed by insurance companies for the purpose of tracking and monitoring the use of a specific vehicle.

Secondly, while the iBBs are primarily installed for insurance purposes, mEDRs are installed for safety reasons. This reflects directly on the amount of data that is collected by each device. The amount of data collected by mEDRs is larger than the iBBs since it collects mostly Driver Data with the purpose of determining the driver’s risk profile, or Vehicle Data, to analyse the correct functioning of the vehicle. Although, the collection of data by both iBBs and mEDRs is subject to data protection regulations, such as the GDPR and other EU vertical regulations that are specific to vehicle data.

Thirdly, currently, the access rights of manufacturers are restricted to mEDR data while insurance companies’ access rights are restricted to iBB’s data they installed since these mechanisms are not interconnected while they can co-exist. Additionally, car owners typically have limited rights over mEDR data which may vary depending on the specific legislation which will be further analyzed.

## 4   Stakeholders and their purposes to access vehicle data

This section will focus on an examination of the interests of three key stakeholders who are interested in accessing the data stored in the iBB or mEDR - consumers, businesses, and public bodies. For each of these categories, the interests of both the single, first-party entities – such as a consumer who owns an insurance contract or a car manufacturer–, and third-party entities – for instance, consumer associations or rival insurance companies – will be evaluated.

### 4.1   Particular data and aggregated data

In the context of stakeholder engagement, the data types that will be examined come in two distinct forms: personal/individual, which will be referred as "particular data," and aggregated data. Namely, Particular data refers to information that is useful only in the context of a specific instance, such as a specific person, vehicle, or contract. For instance, the consumer data associated with iBB is essential in its individual form for the consumer, insurance companies, the car manufacturer, and other stakeholders involved in that specific instance. Aggregated data, on the other hand, refers to information that is beneficial when analyzed in a collective form, typically for statistical or evidential purposes. This may include collections of anonymized mEDRs datasets that are used for traffic management or consumer class actions against a faulty car manufacturer.

### 4.2   Granularity and quality of datasets

The quantity of data necessary to fulfil the demands of each stakeholder can fluctuate based on the particular circumstances at hand.

When considering data quality and granularity in the context of GDPR’s portability principle and the principle of informational self-determination, ^7^ it is important to recognize that the right to access data has subjective connotations. For instance, under Article 20 of the GDPR, individuals have the right to selectively port data based on their subjective preferences ^10, 11^. However, exercising this right to its full extent without considering the broader context can lead to adverse outcomes. For instance, if an individual chooses to port only their driving performance data but not data related to alcohol detection, it could create an imbalanced picture of their overall risk profile as a driver, resulting in subjective and distorted reality and reintroducing information asymmetries ^12^ that data recorders are supposed to reduce ^2^.

Similarly, there may be situations where an individual wants to sell a car embedded with mEDR data related to malfunctions, but only selectively ports certain data, potentially compromising the safety of the next owner. Thus, it is necessary to find a solution that balances the individual’s right to informational self-determination with considerations of good faith and fairness towards the recipient of the ported data, as well as public safety.

In addressing this issue, the concept of data quality is crucial. If data quality is understood in GDPR terms as the accuracy of data, ^8^ and based on standardized metrics, then selectively ported data may still be considered accurate and qualitatively sound. However, if data quality is defined as accuracy in describing truth, then porting all relevant data may be necessary in some cases. Ultimately, a nuanced approach is needed to ensure that the right to portability is exercised responsibly and in a manner that does not compromise important interests such as safety and fairness.

It is worth noting that recent policy developments in the concept of portability have shifted from a right to selectively port personal data, ^9^ to more open forms of system interoperability. ^10^ When the shift is materialized in the text of the Data Act and the Common European Data Spaces, it might address the issue of distortion by reducing subjectivity and promoting greater transparency and accuracy, therefore quality, in the data being shared.

### 4.3   Further dses of EDR data

The following section will focus on a non-exhaustive analysis of the reasons why each of the above-mentioned stakeholders may have an interest to access or in further using particular and aggregated data collected by both mEDRs and iBBs.

***4.3.1   Individuals: consumers and collectives*.** For individuals, accessing mEDR data can provide valuable information about a car’s history and potential issues. For example, an individual as the consumer may choose those own mEDR data that depict a ’good driver’ picture, and transfer them to their next insurer in order to obtain a lower premium. Additionally, when considering purchasing a second-hand car, a consumer may access the mEDR data of the vehicle to determine whether it has been in accidents, required significant maintenance, or is subject to potential malfunctions. This information can inform the consumer’s decision-making process and potentially be used as a bargaining tool in price negotiations. It is important to note that in this scenario, the data is being accessed by an individual other than the original car owner.

Consumer associations can also make use of aggregated EDR data in a number of ways. For example, EDR data can be used to initiate legal action under National Consumer’s Law for the recall and withdrawal of faulty cars or car parts (f.i., in Italian law). European class action lawsuits may also be informed by aggregated EDR data, providing evidence of widespread issues that affect a large number of consumers. Additionally, consumer associations may analyze EDR data to investigate whether anticompetitive practices, such as the use of a specific brand of tires, are in place. This information can be used to advocate for fair competition and protect consumers from potential harm.

***4.3.2   Businesses: insurance providers and car manufacturers*.** The use of EDR data by businesses is measured on a carefully balanced scale, while there is a commercial need for the businesses to access EDR data and the businesses who primarily collect, organise and process such data have *sui-generis* database rights to such data, it is imperative for businesses to not only respect the fundamental right to privacy and data protection but comply with industry standards when it comes to deploying privacy-preserving techniques (PPT). The use of appropriate privacy preservation techniques is crucial for the protection of personal data while carrying out data processing, especially in cases where the end result is focused on using such data processing to create training and validation datasets for artificial intelligence (AI) -based functions. It is crucial to appreciate the delicate balance between the fundamental right to data protection enforced through the use of PPTs and the subsequent Fundamental Freedom to Conduct a Business enshrined in Article 16 of the Charter, which is directly impacted by the intellectual property rights and protections awarded to businesses. Therefore, once again, delicate need to balance the Fundamental Rights and Fundamental Freedom emanates, as an overarching focus on either the fundamental right to data protection or the fundamental freedom to conduct a business adversely affects the other. For instance, *sui generis* databases (as discussed before) that utilize personal data as the content they collect and organize, may be protected through intellectual property rights, however, this does not exempt the creators of such a database from adhering to personal data protection laws. However, at the same time, the data protection regulatory framework does not bar the creation of a *sui generis* database based on validly collected and processed personal data ^13^. However, in the event such a balance between Intellectual Property Rights and Data Protection rights were not maintained, it would create a chilling effect on both the Fundamental Right to Data Protection as well as the Fundamental Freedom to Conduct a Business, therefore leading to either an economic gridlock or the rampant violation of the Right to Privacy in the garb of exercising intellectual property rights.

There is a wide array of businesses to which EDR data is of crucial importance, however for the purposes of this paper, the investigation is limited to insurance businesses and businesses oriented around car manufacturing (including businesses involved in the refurbishment of vehicles).

**Insurance providers:** The processing of EDR data offers insurance providers several opportunities to enhance their operations and decision-making processes. By leveraging this data, insurers can achieve various objectives ^14^.

Firstly, insurance companies can utilize EDR data, which includes information about driving styles and the number of drivers associated with a specific vehicle, to calculate premiums more accurately. This data allows them to assess driver profiles and determine risk levels more effectively ^15^.

Furthermore, EDR data can serve as valuable evidence in legal proceedings, enabling insurance providers to contest claims of insurance denial. The data can be presented in court to support or refute allegations made by policyholders. Another advantage of EDR data is the ability to distinguish between regular wear and tear on a vehicle and damages resulting from accidents. By analyzing the data, insurers can evaluate the impact of these factors on vehicle performance and determine the appropriate coverage and claim settlements accordingly ^16^.

Moreover, insurance companies can identify market trends by analyzing the evolving driving data derived from EDRs. By detecting patterns or shifts in specific technical issues that suggest foul play, insurers can adjust their policies to exclude coverage for damages related to such issues, thereby mitigating potential fraudulent claims

In addition, EDR data enables insurers to create tailored insurance policies based on the number of drivers and behavioural data collected. If the data reveals multiple drivers for a particular vehicle, the insurance provider can adjust or suspend certain features of the coverage to align with the associated risks. Furthermore, insurance providers can leverage EDR data to evaluate the risk profile of potential new customers efficiently. This data allows them to assess the driving habits and behaviour of individuals without requiring lengthy profiling processes, enabling faster underwriting decisions.

Lastly, access to aggregated EDR data from vehicles of the same brand and make empowers insurance providers to evaluate if specific car models exhibit manufacturing problems that increase the risks of accidents. This knowledge can guide insurers in adjusting their pricing, coverage, and risk assessment for these particular models.

**Car manufacturing companies:** The EDR data holds crucial significance in the car manufacturing industry, as it offers valuable insights that find multiple applications.

These applications include tracking vehicular performance, monitoring car servicing timelines based on recorded wear and tear, and providing emergency services in cases of accidents or unexpected breakdowns.

Additionally, the data helps in detecting warranty tampering of the engine and other car parts, as well as calculating repair and refurbishment costs. It also serves as a valuable resource for research and development purposes.

Furthermore, the EDR data can be utilized as evidence in legal proceedings, particularly in lawsuits involving claims related to manufacturing defects.

Lastly, it facilitates swift resolution of technical issues when the car manufacturing company employs hardware or software components similar to those experiencing malfunctions in vehicles from other manufacturers.

***4.3.3   Public bodies*.** With the digitization of governments and the interconnection of public databases, public sector bodies can benefit greatly from the use of EDR data, especially vehicle data and else data both in aggregated and particular forms. The use of such data can result in substantial cost savings for society, avoiding repeated collection that could generate the same results, and extracting more value off of the same data for public purposes, such as traffic management since the public sector is not able to collect this amount of data by itself.

Therefore, the next sections will explore potential uses of EDR data by the public sector.

As previously mentioned, EDRs are devices installed in vehicles to record technical information for a brief period before, during and after a triggering event, which is normally a car crash. Taking inspiration from the United States, Section 2 (4) of the Driver Privacy Act of 2015, EDR data can be used for the purposes of determining the need for, or facilitating, emergency medical response in response to a motor vehicle crash. According to the US Center for Preparedness and Response (CDC), EDR data can be an essential tool to assist emergency care partners to prioritize goals and objectives that advance the use and geographic coverage of current vital records systems to improve vital records data timeliness, quality, and access. ^11^ Thus, it is understandable that the data from EDRs can be used by the public sector to facilitate the management and planning of emergency medical personnel adapted to specific areas.

Municipalities, especially the ones that adopt the name of ’smart city’ often want to improve municipal traffic management through the collection and use of data. Taking inspiration from Montana’s regulation on Recorded Data ^12^, EDR data might be retrieved or used in for the purposes of improving traffic management with the condition that the identity of the owner or driver is not disclosed in connection with that retrieved data.

As Abdulhafedh points out, this data might be valuable for the making of a traffic management plan, which can include data from vehicle accidents that have occurred in a specific area. With this data it is possible to track if the road or the traffic environment contributed to the accident, shaping future public policy on road management and future designs of a safer road infrastructure ^17^.

The introduction of event data recorders storing a range of crucial anonymised vehicle data, accompanied by requirements for data range, accuracy, resolution and for its collection, storage and retrievability over a short period before, during and immediately after collision (for example, triggered by the deployment of an airbag) is a valuable step in obtaining more accurate, in-depth accident data. All motor vehicles should therefore be required to be equipped with such recorders. These recorders should be capable of recording and storing data in such a way that the data can only be used by Member States to conduct road safety analysis and assess the effectiveness of specific measures taken without the possibility of identifying the owner or the holder of a particular vehicle on the basis of the stored data.

The most common exception for the retrieval or use of EDR data is for the purpose of an investigation. As Hampton *et al.,* points out, the use of EDR data has the potential to provide an independent measurement of crash severity allowing an easier accident reconstruction for the purposes of investigations ^18^. Automobile event data recorders hold data that is crucial to court proceedings and can be a reliable source of evidence for court proceedings related to collisions ^19^. In United States legislation, this exception is located in the Driver Privacy Act of 2015, Section 2 (3). As previously mentioned, so far 17 states in the United States ^13^ have enacted statutes relating to event data recorders and privacy, while in others although the topic has not been legislatively addressed, interesting Court rulings were issued on the topic of EDR and privacy. ^14^

In the case of investigations, when a court orders the retrieval or the use of data or within the context of a investigation, the data from the EDR might be used, even without the consent of the vehicle’s owner.

When it comes to the EU, EDR regulation came later, in 2019 with Regulation (EU) 2019/2144. However, contrary to the US legislation, according to Article 6, (4) d) EU EDR data collection is limited to the purposes of accident research and analysis not liability in cases of vehicle accident investigations. Therefore, the US EDR federal legislation is broader and has more application possibilities than the EU one.

The public sector also may access EDR data for the purposes of vehicle safety research; this is an essential use for this data since traffic accidents are one of the major causes of death in the world. For instance, EDRs provide accurate data on driver’s use of seat belts during vehicular crashes, data that is more reliable than drivers’ statements. Through the use of this data, it is possible to conduct research on the use of seat belts and skew estimates of the effectiveness of seat belts in reducing fatalities and injuries ^20, 21^. This is the main purpose of the EU Regulation (EU) 2019/2144 which has its main focus on accident research and analysis.

EDR data also can be valuable data for statistical purposes, through the aggregation of data, statistics can be made on traffic accidents. Statistics can be made by the Municipal Data Office (based on Article 15(c) of the Data Act Proposal and Recital 162 GDPR), by the Member State Statistical Office (based on Member State Statistical Regulation) and also by the Statistical Office of the European Communities (based on the EU Regulation 223/2009). Statistics on vehicle accidents also fall under the scope of the EU Regulation (EU) 2019/2144, since its main focus is accident research and analysis.

Following this non-exhaustive analysis of the possible interests of each stakeholder to both access and port data, in the following sections, this study will focus on the legal analysis which will either permit or block data access.

## 5   Legal bases to access vehicle data: Legal enablers *De Lege Lata*

The present section will concentrate on the analysis of the legal mechanisms that might enable the access and use of mEDRs and iBBs data according to the interests presented in the previous section. To do so, this section will be divided into three main horizontal regulations regarding data, namely the General Data Protection Regulation (GDPR), the Free Flow of Non-Personal Data Regulation (FFNPD), and the Open Data Directive (ODD).

These already existent legal mechanisms allow certain interested parties, under specific circumstances, to access particular types of data, as long as the data is processed according to established rules. Yet, the sheer number of stakeholders, available data, and potential use cases have resulted in a massive backlog of unfulfilled requests under the current access regimes–which begged for the ideation of the Data Strategy.

Therefore, to assess the effectiveness and efficiency of the current legal framework and realize the full potential of the Data Strategy, it is crucial to analyze the scope of the data access gap, including the existing access rights and possible obstacles that hinder the reuse of data. By doing so, insights can be gained into what measures might be taken to bridge the access gap and facilitate the secure and responsible sharing of data.

In this analysis, any use of the EDR data that goes beyond that for which the data was collected is by definition a "further use". When the purpose for the use of the data changes, so does the legal basis for its processing.

### 5.1   GDPR

***5.1.1   Individuals in GDPR*.** This subsection aims to identify provisions within the GDPR that grant individuals access to data stored in iBBs or eMDRs that they would not otherwise be able to obtain. Such data may include an individual’s own personal data, which is referred to as a ’Subject Access Request’, or incidentally to personal and non-personal data of third parties. The term ’incidental access’ refers to situations where a requesting party seeks access to a personal dataset that is inherently interconnected with the data of others. In these scenarios, conflicting protection mechanisms come into play, granting rights to both parties involved, including the data protection rights of individuals whose data is intertwined. Nevertheless, access may still be permitted if a careful balancing of rights determines it to be justified.

Regarding access to aggregated data, if such data has been anonymized, it is no longer classified as personal data. Therefore, individuals may have access to such data without infringing upon GDPR regulations ^22, *contra,*^.

Taking a high-level view, the expansive definition ^23, 24^ and broad interpretation of personal data can be viewed as a meta-mechanism for individuals to gain access to information held by third-party private (business) or public (municipality) entities, provided that the individual can be identified through such information. If the definition or interpretation of personal data were narrower, such as including only a person’s name, surname, and address, or sensitive data, then access to data held by third parties would be limited to only such evidently personal data, while excluding other types of data, such as observed or inferred data.

According to Article 6,1(f) (Legitimate Interest to Access) the processing of personal data is considered lawful if it is necessary for the legitimate interests of the data controller or a third party, as long as those interests do not override the rights and freedoms of the data subject, especially when the data subject is a child. The legitimate interests of third parties, in the context of GDPR, can refer to situations where another individual who is neither the data subject nor the controller has a justifiable reason to access personal data. In such cases, the controller may have valid legal grounds to process personal data where it is necessary to fulfil the legitimate interests of the third party.

For example, an individual may have a legitimate interest in accessing the driving records of another person if they are considering renting out their car to someone, such as car dealers who rent cars to Uber drivers. Similarly, if an individual is looking to hire a chauffeur for a family or business trip, they may have a legitimate interest in accessing the driver’s personal data to ensure that they are a safe and competent driver. Here, the primary focus lies not on evaluating whether legitimate interests surpass the three-part test involving purpose, necessity, and balancing with the rights and freedoms of others–which depends on the context. Instead, what holds significance is the acknowledgement that, according to the interpretation of the WP29 and subsequent authorities, nearly any legal interest can be deemed legitimate ^25, 15^ thereby facilitating access.

According to Article 9,2(f) (Access to Sensitive Data to Exercise Legal Claim), data processing may be necessary in cases where it is required for the establishment, exercise or defence of legal claims or when courts are acting in their judicial capacity. The described scenario involves an individual acting in the capacity of a controller and making a request to access another person’s personal data within a BB, citing a legitimate interest, such as gathering evidence to support a civil liability case. Specifically, accessing Driver Data can reveal whether the driver involved in an accident was under the influence of alcohol or drugs. To comply with the principle of data minimization, the recipient of the data should only have access to information that enables them to make such an inference, which in this case is limited to data collected within a reasonable timeframe following the accident. This requirement should also be reflected in the technical means employed, although it is likely that machines already only collect data in the vicinity of the triggering event.

According to Article 15,1 (Right of Access by the data subject) the data subject has the right to request confirmation from the controller regarding whether their personal data is being processed or not. If their data is being processed, they have the right to access that personal data and receive a copy of it. In general, data protection laws give individuals the right to access their own personal data held by a controller. However, as said, there are situations where an individual may request access to his own personal data that incidentally also pertain to someone else. In these cases, the controller must ensure that the data subject’s request meets certain criteria before granting access.

One such criterion is that the requested information must also pertain to the data subject. This means that the data subject shall have a valid reason for accessing the information, such as needing it to prove their own involvement in a particular situation, as in the example given of person A accessing their own data to demonstrate that person B was in the car with them. There are plenty of similar examples in the jurisprudence of the Court of Justice of the European Union, starting with the *Nowak* case admitting the possibility of accessing others’ personal data for the need to access their own. ^16^

Irrespective of the limited range of processing activities available for the personal data of a third party, a controller cannot exclude the data subject’s right of access even if their request is based on subsequent rights, such as the right to erasure.

According to Article 16 (Right to Rectification), the data subject has the right to request that any inaccurate personal information about them be corrected by the data controller promptly. Depending on the reasons for the processing, they also have the right to have any incomplete personal information about them completed, which may involve providing additional information to the data controller.

When a data subject makes a rectification request, the activities related to the personal data belonging to both the data subject and a third party are more extensive than those for a simple access request. While the data can be accessed, in the case of incomplete or inaccurate personal data, some of it may need to be created, updated, or deleted. For instance, incomplete personal data may need to be created, inaccurate data may require updating, and any update may require the deletion of previously stored data.

When one ground of a limited list apply, the data subject has the right to request the deletion of their personal data from the controller without undue delay, and the controller must promptly comply with this request (Article 17 - Right to Erasure).

As established by the Google Spain case, ^17^ individuals have the right to request the erasure of their personal data if the information is inaccurate, inadequate, irrelevant, or excessive for the purposes of data processing. The term ’inadequate’ may encompass situations where the data is no longer relevant, where consent has been withdrawn without alternative legal grounds for the processing, where processing is unlawful, or where valid objections have been raised.

All data, including links to where the data is available, must be deleted upon request. However, exceptions may apply. For instance, the right to erasure may not be exercised if it would infringe on the rights and freedoms of others. For example, in the case of inextricably mixed datasets, the deletion of personal data may also result in the deletion of other individuals’ personal data or other legitimate business data that has been collected and processed.

Article 20 GDPR (Right to Personal Data Portability) has the primary purpose to empower consumers of digital services by preventing lock-ins, facilitating data subjects in the process of switching providers and multi-homing "without hindrance" ^26^. It does so by allows individuals to "receive" their personal data, and "transmit" it to another controller. To ensure data interoperability between systems and services, the dataset of personal data must be downloadable in a structured, commonly used, and machine-readable format.

Experts in the field claim that the Data Portability right can be used in the context of vehicle data ^5, p. 19^. If the circumstances so allow, the individual has the right to request the original controller to directly transfer the data to a new controller. However, the ease of such transmission is contingent upon certain datasets. Technical limitations may arise where direct porting would impede service functionality, while economic limitations may occur if fulfilling the request proves excessively costly for the controller. Finally, so called ’legal limitations’ relate to the rights and freedoms of others, especially when third-party data is involved. In cases where businesses have invested resources in the acquisition, transformation, or analysis of personal data belonging to the data subject or other individuals, porting of, for instance, filtered pictures or collages made with proprietary software, private conversations or group pictures may pose challenges. Therefore, the chance of porting third-party data will be determined on a case-by-case basis.

**Restrictions and exceptions** The GDPR provides certain exceptions outlined in Articles 85-89. With a valid legal basis, these exceptions permit controllers to process the personal data of a data subject under certain circumstances, such as for exercising their freedom of expression and information for journalistic, academic, or artistic purposes as outlined in Article 85. Additionally, personal data contained in official documents held by public bodies may also be made public to ’reconcile public access’. In such cases, personal data protection rules safeguard the data of others. These rules create a protective barrier around personal data, making it more difficult for third parties to access and use such information. However, for those seeking access to personal data, these exceptions serve as a means to obtain such information.

Importantly, the exceptions outlined in Article 89 can serve as access points, allowing third parties to penetrate the protective shield surrounding personal data, but only under certain safeguards. Article 89 of the GDPR stipulates that processing personal data for archiving purposes in the public interest, scientific or historical research purposes, or statistical purposes must be subject to appropriate safeguards. These safeguards are technical measures that ensure data minimization is observed, such as the use of pseudonymization or anonymization techniques whenever possible. In addition, national laws can include derogations that limit the rights of data subjects for the purpose of public archiving (Articles 15, 16, 18, 19, 20, 21), or for research and statistics (Articles 15, 16, 18, 21).

Article 23 of the GDPR allows for restrictions to be placed on the rights of data subjects or the obligations of controllers by law when necessary, provided that the restriction respects the essence of the rights and freedoms of data subjects. Recital 79 provides further clarification, stating that restrictions may be imposed by Union or Member State law on specific principles and rights, such as information, access, rectification or erasure of personal data, the right to data portability, the right to object, decisions based on profiling, communication of a personal data breach to a data subject, and certain related obligations of controllers. These restrictions are only permissible if they are necessary and proportionate in a democratic society to safeguard public security, protect human life, prevent or prosecute criminal offences, execute criminal penalties, safeguard important public interests of the Union or a Member State, including economic or financial interests, maintain public registers, further process archived personal data for specific purposes, or protect the data subject or the rights and freedoms of others, including social protection, public health, and humanitarian purposes. It is important to note that any such restrictions must be in accordance with the requirements set out in the Charter and in the European Convention for the Protection of Human Rights and Fundamental Freedoms.

Finally, Article 21 (Right to Object to Processing) grants data subjects the right to object to the processing of their personal data based on point (e) or (f) of Article 6(1), including profiling based on those provisions, if the objection is related to their particular situation. The controller must stop processing the personal data unless they can demonstrate compelling legitimate grounds for the processing that override the interests, rights, and freedoms of the data subject, or for the establishment, exercise, or defence of legal claims.

**Technical blockers** In the case of data portability, the law stipulates that the exercise of such data protection right is dependent on the technical capabilities of the data controller. With regard to the Right to Direct Data Portability, as outlined in Article 20 of the GDPR, the ability to exercise it is contingent upon the technical feasibility of transferring data in a machine-readable format. If the data controller is unable to provide the data in a compatible format, the full exercise of the right to portability may not be possible. From the individual data subject’s standpoint, exceptions to the exercise of data protection rights that grant individuals access to their data can be viewed as blockers to such access. However, these same rights can also pose obstacles to access for others, such as data holders willing to aggregate them into larger databases. In such cases, exceptions to the exercise of rights of the data subject become access enablers for third parties.

***5.1.2   Businesses in GDPR*.** The GDPR is a mighty tool when it comes to delineating the contours within which the processing of data must occur. In its provisions, the GDPR alienates the purposes for which the processing of data may be deemed as lawful, Article 6 of the GDPR, which details the lawfulness of processing through a combined reading of its subclauses Article 6(1)(a) and Article 6(1)(c) which state that the processing is deemed lawful if the data subject has provided their consent for processing of data for one or more specific purposes as well as when the processing is necessary for compliance with a legal obligation to which the controller is subject, which in this case is directly applicable to businesses since they are bound by contractual legal obligations of the businesses who are processing EDR data for providing services to their clients. The businesses processing EDR data are free to do so in compliance with the provisions of Article 6 of the GDPR (as detailed above), which discusses the lawfulness principles attached to the processing of personal data. In order to duly process the data, the businesses require specific consent from the data subject coupled with the need to process the data in order to perform the obligations under the insurance contracts such as honouring insurance claims and for car manufacturing businesses to provide breakdown maintenance or car servicing notifications ^27^. It is evident that there are no specific roadblocks in the processing of EDR data and free but regulated access to the same is provided under the provisions of the GDPR. Further, through the provisions of Article 9 of the GDPR, car manufacturing businesses, through the EDR data can act as a link between their customers and emergency responders [ **?**] in case of accidents and therefore, the processing of special categories of personal data is allowed under Article 9(2)(c) of the GDPR. Further, Article 9(2)(f): Although, EDRs are seldom found to include special categories of personal data, the use of EDR data will be legally allowed under the GDPR if such data has to be used in order to indicate a certain fact or behavioural pattern in courts (as illustrated in the use cases held in Section 3.2.2 where insurance companies and car manufacturers may use EDR data as evidentiary support in a court of law).

Contrary to provisions which illustrate the enablement of processing EDR data by Car manufacturing companies and businesses, the GDPR through its provisions also lays down a set of reasonable restrictions when it comes to the processing of personal data which may be held in the EDR dataset. Transparency has been a central feature of the EU data protection law, it is about engendering trust in the processes which affect the citizen by enabling them to understand, and if necessary, challenge those processes. As a part of the provisions of the GDPR under Article 5(1)(a) in addition to the requirements that data must be processed lawfully and fairly, transparency is now included as a fundamental aspect of these principles. ^18^ Further, Article 5(1)(c) which harbours the data minimization principle might also be invoked as a technical blocker when it comes to the processing of EDR-based personal data by businesses ^28^. Further, Article 17 of the GDPR provides a data subject with the Right to be forgotten and in turn, the right to obtain from the controller the erasure of the personal data, therefore, requiring the personal data contained within the EDR being processed by the businesses to be removed from the dataset, required to create insights ^29^. The GDPR also lays down a few restrictive rights such as the Right to restriction of processing under Article 18, this provision allows for the data subject, in the present case the consumer to allow for the restriction of processing their personal data in the event, one of the conditions laid down in the provision of Article 18 is met. In the present case, in the event the consumer decides against the use of services of a particular business, they may require a restriction on the processing sing of personal data under Article 18,1(d) ^30^. Additionally, the right to data portability which is contained in Article 20 of the GDPR, allows the customer the freedom of choice to decide to port their personal data to another business and therefore requires the Right to Data portability to be exercised, which would indubitably set into motion a restriction on the processing of the customer’s personal data by the business which is the primary custodian of their personal data and is obligated to port their customer’s personal data to another business at the direction of the customer ^31^. In addition to the right to data portability, the GDPR also provides the data subjects the right to object under Article 21 and in this case, it empowers the customer to exercise their right to object against the processing of personal data based on the grounds held in Article 6(1)(e)or Article 6(1)(f) which, incidentally is also one of the enablers of the personal data processing, under the GDPR ^32^. Lastly, the GDPR under Article 22, which details the legal obligations concerning automated individual decision-making (including profiling), acts as a crucial and indispensable guard-rail against the practice of automated decision-making and empowers the customers whose EDR dataset is being collected to object against being subjected solely to automated decision-making processes fueled through the processing of their personal data ^30^.

***5.1.3   Public bodies in GDPR*.** This segment aims to make an analysis of the GDPR provisions that enable access to personal data by Public Bodies. Due to the multiple legal systems and administrative organisations in the EU, the term “public bodies” is used in a broader sense, which might have different interpretations depending on local understanding.

The analysis of the GDPR was done both article by article but also in a holistic manner which allows this exploration to observe the underlying principles and objectives of the regulation. The first article that is relevant to the analysis is the legal basis for processing which is located in Article 6,1(c) GDPR. Throughout the analysis, it is possible to observe that the public sector might have access to the eMDR recorder driver data for legal obligation purposes if this legal obligation originates directly from Member State law. Thus, in the hypothesis that a Member State enacts a law that obliges the sharing of EDR data with the public sector. It is important to highlight that this law must require a certain processing operation. ^19^ This legal provision must be clear, precise and define exactly that the public sector will process EDR data for the purposes of compliance with legal obligations. ^20^

The second basis for processing which is pertinent is processing for the performance of a task carried out in the public interest located under Article 6,1(e) GDPR. Under this hypothesis, the public sector might process eMDR data to carry out a task in the public interest. Although the concept of ’public interest’ might vary on Member States’ legal landscapes, the nature of the eMDR data is essentially linked to the public interest, such as technical inspections, traffic investigations, road management, and many other purposes. Some of these tasks are already outsourced to private entities, for this reason, this legal basis must be interpreted under a functional approach, even if the controller is a public authority, private entity, or publicly owned entity. ^21^

**Purpose Limitation Principle** under Article 5,1(b) GDPR, can be, under certain conditions, a legal blocker for public sector access to personal data. The underlying rationale under this principle is that personal data can only be used for specific purposes, in a transparent, and predictable manner. Predictability is present when further processing is sufficiently related to the original purpose. When it comes to the use of eMDR data, its primal processing is the collection of driving data for insurance purposes and the secondary processing would be for purposes of analysing driving patterns for purposes such as improvement of traffic management, investigation of vehicle accidents, and other general research purposes. Under this analysis, it is possible to observe a sufficient relationship between the original processing of EDR data with the secondary processing purposes. ^22^

Additionally, this further processing falls under exceptions listed in Articles 5(1)(c) and 6(4) GDPR which are the possible further processing for archiving purposes in the public interest, scientific or historical research purposes, or statistical purposes; further processing allowed or required by Union or Member State Law; further processing for a compatible purpose under Article 6(4) GDPR and further processing based on the data subject’s consent.

***5.1.4   Technical blockers*.** When it comes to legal blockers, it is relevant to highlight that although the GDPR aims to allow the free flow of personal data under a certain legal basis, it also has put in place principles that aim to give data subject control over the use of their data.

According to the principle of data minimisation located in Article 5,1(c) GDPR, data collected and processed should be adequate, relevant, and limited to what is strictly necessary for relation to the purposes for which they were processed. Therefore, in this case, it must be assessed if the processing of personal data is adequate or appropriate, and relevant for the purpose of the processing. Therefore, this principle might be a blocker for further access to eMDR driver data by the public sector since the further processing of driver’s personal data might not be proportionate for the purpose of processing such as traffic management. ^23^

At last, one possible blocker for the further use of eMDR personal data is Article 12 GDPR, which regulates that the data subject has the right to obtain transparent information, communication, and modalities for the exercise of his/her rights. According to this article, controllers must take the appropriate measures to provide any information referred on Articles 13 and 14 of the GDPR. The further processing of data by the public sector also makes the duty of ensuring the rights of the data subject harder, such as the right to erasure. This article is closely related to Recital 60 which poses the obligation to data processors to inform data subjects of the existence of processing operations and their purposes, including the existence of profiling and its consequences.

Further processing of eMDR data also poses a question when it comes to the right of data erasure – under Articles 17 and 19 GDPR – since multiple data processors would have to ensure that personal data are not kept longer than is necessary, making it harder for the data subject to keep track of his or her personal data.

### 5.2   Free Flow of Non-Personal Data Regulation (FFNPD)

***5.2.1   Individuals in FFNPD*.** The lawmaker behind the FFNPD ^33^ identified that two key barriers to data mobility and the growth of the data economy in the European Union were data localization requirements imposed by Member State authorities and vendor lock-in practices prevalent in the private sector. These factors hindered the effective and efficient functioning of data processing and the development of a harmonized internal market for data-related services.

As per Article 2 of the FFNPD Regulation, there are two distinct categories of individuals who may be interested in accessing data from iBBs or eMDRs. These include ’users’ and ’professional users’. The former encompasses a natural person who uses or requests a data processing service. The latter, is to a natural person who uses or requests a data processing service for purposes related to their trade, business, craft, profession, or task.

However, the Regulation primarily focuses on promoting the free flow of non-personal data within the European Union and facilitating the switching of cloud service providers for professional users, rather than on individual access to data.

As the aim of this section is to identify provisions that create access rights to individuals, it is important to acknowledge that the Free Flow of Non-Personal Data Directive does not directly provide such access rights. While the directive may indirectly facilitate greater availability and accessibility of datasets for individuals through its efforts to promote competition and reduce barriers to entry in the cloud services market, it primarily focuses on promoting the free flow of non-personal data within the European Union and facilitating switching of cloud service providers for professional users.

This could potentially lead to a more open and accessible data ecosystem, with more opportunities for consumer organizations to access data for research, advocacy, and other purposes. While the directive does not guarantee access to data for consumer organizations, it sure creates a more favourable environment for such access to occur.

***5.2.2   Businesses in FFNPD*.** There are no observed legal restrictions under the FFNPD regulation which may lead to a restriction in the collection and processing of non-personal EDR data as held by businesses involved in the insurance sector and car manufacturing. Inf act, the provisions of the FFNPD through its Article 6 encourage businesses to develop self-regulatory mechanisms such as codes of conduct for the free flow and regulation of the non-personal data in the EU, thereby strengthening the data portability requirements for the non-personal EDR data held by businesses and established an expansive scope of the right to data portability from personal data (as held in the GDPR) to non-personal data ^31^. This also has a direct impact on the competition within the various businesses which are involved in using EDR datawhich comprises both personal and non-personal data (as explained in section 2.1 of this paper).

***5.2.3   Public bodies in FFNPD*.** The EU Regulation on FFNPD aims to create a borderless EU for the flow of non-personal data, removing obstacles such as data localisation restrictions. Different from the GDPR, the FFNPD only covers non-personal data, which makes the re-use of eMDR data by Public Bodies an easier task. Under Article 5 FFNPD, competent authorities in the public sector have powers to request and obtain access to eMDR data for the performance of their official duties, such as traffic accident investigations and other connected activities. Therefore, taking a holistic interpretation of the GDPR and FFNPD, Public bodies have the possibility of using both personal and non-personal eMDR data. Additionally, following the underlying principle of liberating the flow of non-personal data within the EU, Article 7 FFNPD creates a procedure for cooperation between authorities, facilitating cooperation between different Member States, this cooperation would eventually facilitate the public sector access to vehicular and else data in an easier manner.

Yet, no blockers were found in the analysis of the FFNPD, which facilitates the main objective of this study which is a joint analysis of multiple regulations for the purposes of further use of eMDR personal and non-personal data, referred to as particular data.

### 5.3   Open Data Directive (ODD)

***5.3.1   Individuals in ODD*.** The Open Data Directive (ODD") ^34^ recognizes the interest of individuals, in their various roles as citizens, ^24^ users, end-users, ^25^ consumers, ^26^ and researchers, ^27^ to gain access to public sector information.

By mandating member states to make all existing documents ^28^ reusable, the directive is effectively creating an opportunity for individuals to access public sector information. This opportunity does not amount to an absolute right as it is subject to restrictions and exceptions. ^29^ This notwithstanding, the likelihood of a scenario where a car manufacturer or an insurance company operates as a public undertaking, and is additionally required to disclose certain data under the auspices of the open data directive, is exceedingly low to the point of being negligible.

***5.3.2   Businesses in ODD*.** The Open Data Directive is a regulation aimed at fostering sharing of data held by Public Sector Bodies of the Member States of the EU, therefore, this regulation finds no application in the schematic concerning the flow of data generated by EDRs of private citizens and accessed by private businesses such as Insurance Companies and car manufacturing businesses ^35^. Further, it is noted that the Open Data Directive does not apply to the documents held by companies which hold commercial value such as insights pertaining to business analytics etc. ^36^, therefore, the insurance companies which appear in the EU’s register of insurance undertakings are exempt from application of the Open Data Directive. Finally, the Open Data Directive also finds no applications towards documents which are excluded from access by virtue of the access regimes in the Member State, including on grounds of commercial confidentiality (including business, professional or company secrets). Therefore, owing to the aforementioned reasons it is concluded that the open data directive has no bearing on the sharing or blocking of EDR-generated data and its use by businesses.

***5.3.3   Public bodies in ODD*.** It was not found any legal enabler or blocker to public sector access to EDR data in the Open Data Directive.

### 5.4   Milestone 1 (M1): Examining accessibility of EDR content through existing access regimes

***5.4.1   M1: Individuals*.** The broad interpretation of personal data in GDPR results in the extension of access rights to a substantial amount of data in an EDR dataset. However, the expansion of these personal rights over data amplifies access solely for the individual, while limiting that of others. This is despite the fact that, from an economic perspective, data is a non-rival good ^37, 38^, and that data protection regulation aims to promote the free flow of personal data. The reason for this is that the existence of rights to control the flow of information can, in certain cases, lead to the exclusion of others from accessing the data, while in others, significantly limiting its processing. Thus, as the data within an EDR is finite, any increase in the amount of personally identifiable information (the driver data) contained in the dataset effectively reduces the number of other types of data, such as vehicle data and else data categories, along with the access rights to the same dataset of other interested parties. In the broader context of data access, the data subject has progressively more methods to obtain and modify their driver data, with an increasing ability to have it modified by the controller. These manipulations generally fall under the CRUD classification, with the exception of data portability where the data subject can request that their data be downloaded or transferred directly to a third party–when technically feasible.

The aforementioned analysis is accurate when referring to individuals’ particular data. However, in the case of aggregated data, the information extrapolated from the EDR no longer can be labelled as vehicle data, driver data, or else data, because it loses the characteristics of individuality and identifiability. As such, it can be accessed and utilized by any other relevant stakeholder, for purposes ranging from exercising public tasks to conducting private business, to research, statistics, and so on. Notably, the loss of granularity in data should not necessarily be considered a loss in quality. In fact, the impact on quality can vary depending on the context. For example, in some cases, the aggregation of data can eliminate outliers that may be attributed to data collection errors, resulting in a more refined data set. When EDR data is aggregated, then rules enhancing the free-flow of non-personal data data augment the access regimes, creating a more open and accessible data ecosystem, with opportunities for consumer organizations to access data for research ^39^, advocacy, and other purposes.

***5.4.2   M1: Businesses*.** The combined regulatory framework concerning data access which includes the above-discussed regulations namely the GDPR, ODD, and FFNPD Regulations provides a substantially wide scope for driver data access by businesses engaged in car manufacturing and providing insurance services. The GDPR lays down a regulatory matrix under which businesses such as car manufacturers and insurance providers are enabled to access, update, port and share the personal data (collectively process) which is included in the EDR dataset. Further, the FFNPD Regulations categorically allow for the processing of the nonpersonal data held in the EDR dataset through porting of the personal data and non-personal data respectively.

Finally, the Open Data Directive also finds no applications towards documents that are excluded from access by virtue of the access regimes in the Member State, including on grounds of commercial confidentiality (including business, professional, or company secrets). Therefore, owing to the aforementioned reasons it is concluded that the open data directive has no bearing on the sharing or blocking of EDR-generated data and its use by businesses and such vacuum acts as a data access enabler instead of a blocker in the current situation.

Therefore, upon the combined reading of the abovementioned regulatory matrix, the access regime is focused on providing businesses with unfettered access to both personal and non-personal data contained in the Event Data Recorder.

***5.4.3   M1: Public bodies*.** Following the previous analysis of the enablers and possible blockers in the already enacted Regulations, namely the GDPR and the FFNPD, it is necessary to reaffirm that the analysis of these regulations cannot be done individually, in fact, they are complementary for a full comprehensible analysis. Therefore, under this premise, it is possible to observe that there is already great room for EDR data access by Public Bodies since the GDPR already provides legal enablers that allow the access of personal data without consent when necessary for the previously mentioned purposes. According to Martens ^7^, one such example is the collection of road traffic data. Telecom service operators track mobile phones in cars to obtain important data on road traffic patterns. This tracking is a technical requirement to maintain connectivity between the phones and the network antennas. Therefore, the GDPR permits this data collection without consent as it is necessary for the technical operations of the service.

When transposing this example to EDR data and the public sector, the GDPR is clear in delineating a lawful basis for data access and portability for other purposes that can be considered of public interest which will depend on Member State legislation. Furthermore, the GDPR allows for the onward transmission of this data, provided it is anonymised. In the case of road traffic data, the aggregation process must strip the data of any private phone numbers, ensuring that individual users’ identities remain protected ^7, p. 18^.

If not, this processing might be blocked by general principles of the GDPR, especially the principle of data minimisation since the use of drivers’ personal data might be considered disproportional for the mentioned purposes. Additionally, the further reuse of personal data might also make it difficult for data subjects to exercise their rights such as the right to erasure.

## 6   The proposed Data Act and the expansion of access regimes: Legal enablers *De Lege Ferenda*

Following the general conclusions of possible mechanisms for data access and portability by each stakeholder, the next section will focus on the Data Act (DA) proposal, which aims to expand data access beyond the above-examined regulations, especially for individual and public sector stakeholders.

The proposed Data Act is a legal solution put forward by the European Commission to unlock the potential of datadriven innovation in Europe ^40^. The Act aims to address the reality that data is a vital component of the digital economy, but much of it remains unused or concentrated in the hands of a few large –American—companies ^41^. To unlock this potential, the Data Act provides opportunities for data reuse and removes barriers to the development of the European data economy.

The Data Act facilitates the establishment of governance frameworks and infrastructure, as well as enable data sharing beyond the boundaries of Data Spaces. It is designed to complement the forthcoming European Common Data Spaces (Health, Industrial, Agriculture, Finance, Mobility, Green Deal, Energy, Public Administration, and Skills) which have yet to be fully developed ^42^. Future legislation in relevant areas should align with the broad principles outlined in the Data Act to promote horizontal consistency and coherence across the regulatory landscape. In addition, vertical legislation can set more specific rules tailored to address sector-specific regulatory objectives, allowing for targeted and effective regulation.

One such Common European Space is the Common European Mobility Data Space (EMDS), which aims to enable efficient, safe, sustainable, and resilient transportation by facilitating data access, pooling, and sharing. The EMDS will build on existing initiatives and applications related to transport data and will be supported by efforts to enhance interoperability, security, and the availability and provision of data and services. An upcoming communication will provide information about the EMDS and its role in promoting effective transportation.

Until the European Common Data Spaces are established, the Data Act provides a framework for governance and infrastructure related to data sharing, including outside of the boundaries of data spaces. However, access regimes for data that should be governed within specific data spaces are not yet fully defined, leading to uncertainty in how data access and governance is managed for certain sectors until the corresponding data spaces are operationalized.

### 6.1   Individuals in DA

Chapter II of the Data Act ("Business to Consumer and Business to Business Data Sharing") enhances legal clarity for consumers (and businesses) to access data derived from the products or affiliated services they possess, rent, or lease. In correspondence to such access regimes, manufacturers and designers of digital services are obliged to create products in a manner that facilitates effortless access to data by default, while data holders have obligations to make data available to third parties upon the request of the user. The Data Act is grounded in the level of control that the data holder actually possesses, whether it be *de facto* or *de jure*, over data generated by products or related services.

As expressed in the Explanatory Memorandum, the proposed Data Act expands and complements the right to portability expressed in Article 20 GDPR by granting users the right to access and make available to a third party to *any data* generated by the use of a product or related service, irrespective of its *nature* as personal data, of the distinction between actively provided or *passively observed data*, and irrespective of the *legal basis* of processing. In the context of switching between data processing service covering the same service type, the broadened scope encompasses the portability of ’digital assets’, which refer to "elements in digital format for which the customer has the right of use, including data, applications, virtual machines and other manifestations of virtualisation technologies, such as containers” ^40, Recital 72^.

According to Article 3 (Obligation to make data generated by the use of products or related services accessible) of the Data Act, it is mandatory for products to be developed and produced, and for associated services to be provided, in a way that ensures the data produced through their usage is readily accessible to the user in a secure and convenient manner. Additionally, if it is suitable and applicable, the data should be directly available to the user. Such obligations should normally cover the technical specifications and design strategies thanks to which manufacturers and designers develop mEDR.

The requirement set forth in Article 3 entails that the individual user possesses the right to access any data generated by the use of the product or service, irrespective of its nature, and that such data must be made available. The ’databe-made directly accessible’, or ’direct access’, seems to be something more than its GDPR twin that is a mere reading right: The fact that it is not explicitly categorized within the CRUD classification system highlights that the Data Act expands the individual’s rights by introducing a novel type of action on the dataset. This serves as a clear illustration of how the Data Act extends beyond conventional CRUD operations and incorporates new rights for individuals regarding their data.

The right of users to access and use data generated by the use of products or related services (Article 4( applies to situations where the generated data is not directly accessible by the user from the product, which applies to any data collected in the mEDR that is not shown to the user through an interface. In such cases, the user has a right to immediate, free, continuous, real-time ’data availability’. According to Paragraph 3, the data holder and the user have the option to establish measures to safeguard the confidentiality of shared data, particularly concerning third parties. This provision offers a practical solution to the dilemma presented in Article 20 of the GDPR, which stipulates that the transfer of data must not infringe on the rights of third parties. By enabling the data holder and user to agree on measures to protect the data of third parties, this provision allows for the secure transfer of data, even when it involves the information of others. This scenario may arise in cases such as those where data from the mEDR system contains information about individuals other than the user (Else Data) [note: widening of data access and use!]. The expansion of data access rights is restricted only in cases where the data is used for developing a product that directly competes with the product from which the data was originally obtained. However, this restriction does not apply if the competing product already exists in the market. This provision may be perceived as a limitation on competition, but it is intended to strike a balance between promoting innovation and protecting the rights of data holders.

Article 5 of the Data Act (Right to share data with third parties) establishes a right that complements and expands upon the right to data portability outlined in the GDPR. This right mandates that, upon request by a user, the data holder must *make available* the data generated through the use of a product or related service to a third party without delay and free of charge, at the same level of quality available to the data holder, and in real-time where relevant. Unlike direct portability, this right to ’make data available’ to third parties is not dependent on ’technical feasibility’, making it an unconditional obligation. Moreover, to ’make available’ is technically less complex than to "transfer" data, thereby easing compliance with the obligation of transferring data to a third party. Furthermore, the right is more comprehensive and specific, leaving less room for the controller to evade compliance. As for Articles 3 and 4, also article 5 seems to be fit for addressing data access requests from a consumer to its own mEDR.

Regarding the expansion of the right to data portability, it is important to note that this right is subject to careful scrutiny due to its impact on competition. Both Article 7 and Article 5.2 should be interpreted in this context. Article 7 excludes small and micro enterprises from the obligation to facilitate data portability in certain circumstances, specifically with respect to business-to-consumer and business-to-business data-sharing obligations ^43^. Such exemptions do not apply to data generated by the use of products or related services provided by qualifying micro or small enterprises. Differently, Article 5.2 states that any entity providing core platform services, which have been designated as gatekeepers, cannot be considered an eligible third party for receiving the ported data.

The extension of the data portability right to non-personal data gives rise to a corollary wherein a user requesting data to be ported may not necessarily be a data subject. For instance, this may be the case when a legal entity makes such a request, or when the dataset being transferred contains vehicle data or personal information belonging to a third party. This scenario is acknowledged in Article 5, paragraph 6, which states that "Where the user is not a data subject [...]." This may be considered as an instance of expanding access rights over another individual’s data.

Similarly to Chapter 2, Chapter 6 of the Data Act introduces expanded access rights for individuals, and establishes a set of corresponding rights and obligations. Article 23 (Removing obstacles to effective switching between providers of data processing services), for instance, mandates that providers of data processing services ensure that their customers can switch to an alternative processing service that covers the same service type. Furthermore, since the porting of services must ensure ’functional equivalence’, it is likely that a larger volume of data can be ported under Chapter 6 than under the GDPR, which offers a more limited scope for data portability.

Lastly, addressing cases where the consumer is switching from a data processing service that is not an Infrastructureas-a-Service type and is not compatible with an open interoperability specification or EU Standard, Article 26 comma 3 (Technical Aspects of Switching) provides a right to the Consumer to “export all data generated or co-generated, including the relevant data formats and data structures, in a structured, commonly used and machine-readable format.”

### 6.2   Businesses in DA

The proposed Data Act in Article 3 (Obligation to make data generated by the use of products or related services accessible) states the requirement of product manufacturers which include car manufacturers that fit their vehicles with EDRs to ensure that the data generated by their product is accessible by the user, which in turn may allow for the user to port or share such data with other businesses such as insurance companies, lawyers, other vehicle manufacturers, vehicle service stations etc. Further, it is crucial for us to understand that the Data Act has been created as a ’horizontal legislation’ in order to facilitate data sharing between stakeholders such as businesses (including small and medium enterprises (SMEs)) and customers. The Data Act lays down specific provisions for B2B (business to business) data sharing, B2C (business to customer) and B2G (business to government) data sharing. Article 3 of the Data Act read with Article 5 ensures that the free flow of EDR data is enabled between businesses at the behest of the EDR user or data subject therefore, further allowing businesses to be able to horizontally share data with the various stakeholders ^44^.

Additionally, the data act through its Article 7, which discusses the scope of business-to-consumer and businessto-business data sharing obligations provides the contours within which EDR fitting car manufacturers, primary insurance providers and secondary insurance providers interact with each other when it comes to data sharing and therefore acts as an enabler to data sharing practices in both vertical and horizontal capacities ^44^.

Following suit, the Data Act also enumerates the conditions under which data holders make data available to data recipients and Article 8 discusses the conditions under which data sharing of the EDR data might be operationalised by the businesses at the behest of the customer. These conditions are focused on creating a conducive atmosphere for such data sharing and call for such data sharing practices to be carried out through fair, reasonable, and nondiscriminatory terms and by providing adequate focus to transparency requirements. Further, Article 8 states that a data holder shall not discriminate between comparable categories of data recipients and that the data holder shall not make the dataset available exclusively to a data recipient, unless such a request has been made specifically under the provisions of Chapter II of the Data Act which dictates the rules of B2C and B2B data sharing. This is especially fruitful for businesses which are engaged in providing services for vehicular insurance and allow for data subjects to be able to transfer their data from one data holder to another, this ease of data porting between businesses has a direct and positive impact on not only the competition perspective in the businesses but also allows for data subjects that are the customers of such businesses to benefit ^45^. In its efforts to allow for a more inter-operable and effective data sharing regimen, through Article 23, the data act aims to strengthen the requirements for the semantic interoperability of data amongst various businesses and ensures that the customers can move freely within the market, therefore, promoting competition and ensuring market checks. This provision is directly focused on ensuring that interoperability between businesses, insurance providers as well as businesses and insurance providers is seamless and can help in not only first-party access of EDR data but also third party access to such EDR data without any form of undue hassle ^43^; In keeping with the provisions of Article 23, Article 28 speaks to strengthening interoperability requirements for the EDR dataset within the businesses (car manufacturers as well as insurance providers) per the requests of the customer, or the internal cooperation between companies based on lawful consent of the data subject. This has a direct impact on the free and technically compatible movement of data across businesses ^46^.

Although, not a direct blocking of the use and free flow of EDR data between businesses, the proposed Data Act provides certain conditional provisions which may act as a blockage towards the use of the EDR data by businesses however does wonders for the free market is Article 13 which deals with the unfair contractual terms unilaterally imposed on a micro, small or medium-sized enterprise and this provision aims to balance the interest of parties and liability schematics when it comes to the dealings between established businesses and micro, small or medium-sized enterprise ^46^.

### 6.3   Public bodies in DA

The Data Act is a regulation proposal that was mainly focused on data access by users of connected objects and their software. Nevertheless, the regulation came to light after the COVID-19 pandemic, which had a heavy influence when it comes to sharing private data with the public sector. Therefore the Data Act has a chapter fully dedicated to the topic of business-to-government data sharing.

One of the main legal enablers present in the Data Act proposal is located in Article 14 related to the obligation to make data available based on exceptional need. This article lays out the general ground for business-to-government data sharing under the Data Act, according to it the Public Sector can have access to data upon a specified duly justified request limited in time and scope demonstrating an exceptional need to use the data requested. This special need is further exemplified in Article 15, (a) Data Act in emergency situations, such as climate emergencies, and (b) in other non-emergency circumstances where the public sector body or Union institution, agency or body is "acting on the basis of Union or national law and has identified specific data, which is unavailable to it and which is necessary to fulfil, a specific task in the public interest that has been explicitly provided by law such", e.g., traffic management.

Under these circumstances, the Data Act provides a legal enabler to the public sector would have the possibility to access drivers’ and vehicle data and else based on exceptional need, which can be an emergency situation or non-emergency circumstances in which the public sector body is acting to fulfil a specific task in the public interest. Therefore, the above-mentioned article opens the possibility for the public sector to access data when it is needed for the purpose of a public interest task ^45^.

When it comes to the possibility of public bodies, EU institutions, agencies, or bodies exchanging data obtained with another public sector body, Article 17, 4 Data Act requests However, the data obtained pursuant to this purpose shall be only used for the purpose specified in the request, a section that connects with the GDPR principle of purpose limitation, already analyzed in this study.

When it comes to Article 22 of the Data Act, it also is very much connected with the previously analyzed FFNPD since it regulates that Public sector bodies and Union institutions, agencies, and bodies must cooperate and assist one another, in case of data exchange, facilitating data access by other public sector bodies.

### 6.4   Milestone 2 (M2): Examining the scope of data access enabled by the Data Act

The Data Act aims to provide a framework for governance and infrastructure related to data sharing outside of the scope of data spaces. However, as the European Common Data Spaces are not yet fully developed, there may be uncertainty in the access regimes for data that should be governed within them. Each data space is designed to address specific critical sectors, and until they are fully established, the governance and management of data access may not be fully defined. The Data Act represents an important step towards establishing a framework for data sharing, but further work is needed to fully realize the benefits of the European Common Data Spaces.

### 6.5   M2: Individuals

One could view the Data Act as a substantial amplification in agency, range, extent, and depth of the Article 20 of GDPR, or colloquially speaking, as data access, re-use, and portability ’on steroids’. Under the proposed framework, all stakeholders, including B2G (business-to-government), B2B (business-to-business), and others, possess expanded entitlements to access each other’s data. Interpreted in light of EDR data, this implies that every business-to-anything (B2X) relationship constitutes an extension of access. The rights to access, use and share data under the Proposal are likely to encompass entities beyond the data subjects, such as commercial enterprises, subject to the legal status governing the usage of the device in question. Moreover, As the definition of ‘user’ encompasses legal persons, in case of the exercise of this right by a business, this takes the form of a commercial obligation for the manufacturer/data holder to provide access to data to businesses and allow its exploitation, rather than the individuals’ ‘right’ to access and port their personal data. In fact, according to the concept of ‘user’ adopted by the Proposal, individuals become entitled to enhanced portability right only incidentally, depending on the legal title under which they use the product or the related service (ownership, rental or lease) rather than on their relationship with the information concerning their private use of the product or service ^47^. As for individuals, thanks to the concept of ’user’ embraced by the Proposal, individuals’ enhanced right to portability arises, but only ’incidentally’, depending on the "legal title" they are using rather than on their relationship with the data ^47^. According to Recital 31, users have a right "to access and make available to a third party any data generated by the use of a product or related service, irrespective of its nature as personal data, of the distinction between actively provided or passively observed data, and irrespective of the legal basis of processing”, the expansion of access for the user can be appreciated under two perspectives: on the one hand, the user can access data that was observed by the product or service, concluding a long scholarship debate on whether the concept of data ’provided’ by the user also encompassed data passively ’observed’, or even ’inferred’ by the provider ^10, 11, 48^. For each stakeholder, the expansion in the range of data encompasses driver data, vehicle data, and ’else data’. Additionally, with regards to switching cloud providers, users (both individuals and commercial entities) are entitled to port their "applications" and "other digital assets" along with their data (Article 23), as well as "virtual machines and other manifestations of virtualisation technologies" (Recital 72).

Article 35 (Databases containing certain data) should also be regarded as an advancement in access and reuse. It specifies that the *sui generis* right outlined in Article 7 of Directive 96/9/EC (Database Directive) does not apply to databases that hold data obtained from or produced by the utilization of a product or a related service. This provision explicitly aims to prevent impeding the exercise of users’ right to access and utilize such data under Article 4 or their right to share such data with third parties under Article 5.

Ultimately, the Data Act’s emphasis on technical interoperability underscores a shift away from expanding stakeholders’ access regimes subjectively and towards enhancing the overall technical accessibility into the information systems, and legal accessibility into the market for the information economy.

In comparison to the GDPR (Art. 9,2 ; 15,1 ; 16 ; 17 ; 20) the combination of articles 4, 5, 6 and 26 of the Data Act gives the user (individual and business) extended access and control to the EDR dataset by (1) extending the CRUD actions to "direct access", "get availability"; (2) bolstering the CRUD actions with "immediate availability", or "including relevant data formats and data structures"; (3) extending the access to third-party businesses; (4) giving access to all dataset, (5) first- and third-parties data; (6) and to vehicle data.

### 6.6   M2: Businesses

The Data Act is a horizontal, multi-faceted and multi-stakeholder-oriented legislation, which is a boon for data sharing across businesses, customers and governments. A central feature of the Data Act is its focus on not just awarding the right of data portability across data subjects, data holders and data recipients but also its keen focus on the fairness, transparency, semantic interoperability, non-discrimination and reasonableness of data portability requirements through legal obligations and contractual requirements. Therefore, in terms of EDR data access, the Data Act champions the cause of carving out adequate access regimes of the EDR data and their free flow between businesses, from consumers to businesses and also from businesses to governments, therefore, pairing well with the regulatory regime crafted under the GDPR as well as the FFNPD Directive, and adequately covers the interoperability requirements for both personal and non-personal data, this heterogeneous mixture of personal and non-personal data is also the composition of the EDR dataset and therefore, when examined through the perspective of access regime ^45^.

Additionally, insofar as the definition of ’user’ includes legal entities, if a business exercises the right to portability under Recital 31, it imposes a commercial obligation on the manufacturer/data holder to grant access to data to businesses and permit their exploitation ^47^.

The combinations of the provisions of the GDPR (Article 6,1; 9,2) and Articles 5, 7, 8, 13, 23, and 28 of the Data Act give the business) extend access and control to the EDR dataset by: (1) extending the CRUD actions to "direct access", and "get availability"; (2) bolstering the CRUD actions with "immediate availability", or "including relevant data formats and data structures"; (3) extending the access to third-party businesses; (4) giving access to all dataset, (5) first- and third-parties data; (6) and to vehicle data.

### 6.7   M2: Public bodies

The Data Act Proposal is the latest regulation proposed by the European Commission, which is a part of the European Data Strategy. Its analysis must be done in tandem with the other regulations, especially GDPR and FFNPD. Therefore, when analyzing all the regulations in a holistic manner it is possible to comprehend that there is a legal landscape that enables the access and portability of both driver’s personal data and non-personal data for the public sector bodies. Although the Data Act has its main focus on individual interests in assessing data from connected objects, Chapter V clearly opens an avenue that allows the Public Sector to access meaningful data for fulfilling tasks in the public interest.

Nevertheless, the concept of fulfilment of a task in the public interest has been under modification along the legislative process of the Data Act, and might be transformed into a legal blocker depending on how the legislative process of the Data Act evolves. Although this concept can change depending on the Member State in question, the EDPS-EDPB raised concerns on their joint opinion of the data act, due to the possibility of public sector abuse. Additionally, the industry raised questions regarding this open concept, which might result in further modifications. For instance, the most recent modifications by the Swedish presidency have addressed these points by detailing public tasks on the Recitals. ^30^

Ultimately, Articles 14 and 15 of the Data Act give public bodies enlarged access to EDR data for the purposes of "tasks in the public interest" by (1) extending the CRUD actions to "access", "use"; (2) bolstering the CRUD actions with "including metadata"; (3) extending access to the public sector; (4) giving access to non-personal data, (5) first parties data; (6) and to vehicle data.

## 7   Discussion

While this paper has explored several important aspects of the access, reuse, and portability of data, there remain certain topics that have not been addressed in detail. These topics represent promising avenues for future research and could shed further light on the complexities and nuances of this subject.

**Market for EDR data** Typically, changes in the landscape of providers of prime materials within a market often result in a significant shift in the power structure of the market’s actors ^49^. Similarly, it is expected that the shift in production and ownership of vehicle data from insurance companies to car manufacturers will have an influence on the market for this category of information. In the words of Martens and Mueller-Langer, “even minor shifts in these markets may re-route billions of euros into other hands” ^5^. In the past, insurance companies held exclusive access to data, which gave them the power to set monopolistic prices for selling or retaining it for their own use. However, with the introduction of Event Data Recorders (EDRs) by manufacturers, alternative sources of data are now available, reducing exclusivity and lowering market prices. Additionally, the connectivity of vehicles to the Internet means that some data that was previously only accessible through in-vehicle EDRs is now also accessible by Internet Service Providers (ISPs). As a result, the availability of alternative data sources has reduced monopolistic rents, as noted by Martens ^7, p. 18^. Indeed, as evidenced above, the transfer of control over vehicle data to car manufacturers also implies a shift in the balance of power to broker this data, in a manner that has not been witnessed previously. This shift comes at a time when the law has broadened the access regimes to include other stakeholders who may have an interest in vehicle data. As a result, the industry faces a complex and evolving landscape, where legal frameworks and technological developments will continue to shape the conditions of access, ownership, and control over vehicle data. In this context, industry players must remain attentive to emerging trends and regulatory developments, as they navigate this rapidly evolving environment. Ultimately, the outlook for EDR data is uncertain, given the lack of clarity on whether insurance companies will require the installation of their proprietary black boxes or demand access to the event data recorders held by manufacturers. Furthermore, the conditions dictating such access, whether through contractual agreements or legal directives, are yet to be fully defined. As a result, the future trajectory of the vehicle data market remains uncertain, with industry stakeholders awaiting further regulatory guidance and market developments to shape the landscape.

The previous discussion raises an important point: changes in data access rights can have significant economic consequences for individuals, firms, and society as a whole. While the push for data sharing may seem beneficial when accessing other people’s data, it is important to consider the implications for one’s own data when extracted "against his will and at a cost to him” ^7, p. 20^. If this is read in the context of personal information and the holder of information is the individual, then the European Data Protection Supervisor (EDPS) and European Data Protection Board (EDPB) joint opinion begins to appear more logical. They have called upon the co-legislators to impose constraints or limitations on the use of data resulting from the use of a product or service by any entity other than the data subjects, particularly relevant if the data can lead to accurate inferences about the private lives of data subjects or pose a significant risk to their rights and freedoms. The EDPS and EDPB have proposed the implementation of explicit limitations regarding the use of such data for direct marketing or advertising, employee monitoring, insurance premium computation, and credit scoring ^47^, but it is worth studying whether such limitations would in practice solve the issue.

**Competition and the European Commission’s visible hand** Another interesting point of discussion pertains to the expansion of the right to data portability in the Data Act, which warrants careful consideration due to its potential impact on competition. In this context, Articles 7 and 5.2 are of particular relevance. Article 7 exempts small and micro enterprises from the obligation to facilitate data portability in certain circumstances, such as business-toconsumer and business-to-business data sharing obligations. This is a common strategy employed by legislators to reduce barriers to entry for smaller businesses, similar to creating an entry point at the bottom of the market. In contrast, Article 5.2 proposes a less common approach in this context. It stipulates that entities that provide core platform services, designated as gatekeepers, cannot be considered eligible third parties for receiving ported data. Consequently, this provision does not remove barriers to entry but rather creates them. This can be likened to imposing a ceiling on the top of the market. These provisions have political implications and seem to make the otherwise invisible hand quite visible. However, it should be noted that even with these provisions, dominant players may still find ways to gain an upper hand. As such, it is crucial to read them in conjunction with the Data Strategy and the Digital Markets Act to understand their intended purpose of targeting specific companies.

**Quality, informational determination, and perception** One important aspect of data portability is the extent to which the contractual power of insurance companies may impact an individual’s right to port their data, particularly in cases where the insurance company may refuse to accept only a portion of the personal data related to driving or require access to all of it. While the theory of informational determination supports the right of the data subject to port as much data as they wish, the practical implementation of this right in the insurance industry is still uncertain. In such a scenario, although it may be technically legal that the data subject chooses to port only certain data and leave out others, this approach could lead to a distorted perception of reality and potentially create issues and risks for other parties.

**A Copernican revolution in data sharing?** While discussing GDPR and other existing legislation, Martens notes that "such initiatives are bouncing back and forth between two poles: (a) offering more exclusive rights, either for the protection of personal rights or as an incentive to invest in data collection, and (b) making data more widely available and accessible to facilitate the extraction of new insights from data” ^7, p. 19^. However, what seems to be happening is a fundamental shift in the strategy for access and re-use of data, a Copernican revolution from subjective access to systemic accessibility. Ultimately, the Data Act’s emphasis on technical interoperability underscores a shift away from expanding stakeholders’ access regimes subjectively, towards enhancing the overall technical accessibility into the information systems and legal accessibility into the market for information.

### 7.1   Limitations

Our study is constrained by the selective focus on specific legislations, including GDPR, Free Flow of Non-Personal Data Regulation, and the Open Data Directive. Several other regulations, such as the Type Approval Regulation 2018/858, Digital Content Directive, and the Data Governance Act, could also be relevant but were intentionally excluded for practical reasons. Firstly, the inclusion of these additional regulations would have resulted in an excessively lengthy paper, making it challenging to maintain focus and readability within the scope of our project and time constraints. Secondly, our primary objective was to establish a procedural framework for evaluating the effectiveness of data strategy using the selected regulations as illustrative examples. While considering these omitted regulations might enrich the study, it could potentially lead to redundant content, hindering the overall narrative flow. As a result, the authors opted to maintain a rigorously structured approach, prioritizing precision and clarity over narrative style.

The research partially relies on the EU Commission’s proposal for the Data Act text, as the European Parliament and the Council have recently reached a political agreement pending formal approval by legislators. The absence of the final approved text limits the comprehensive evaluation of our results. Future research will be necessary to compare the proposed and final texts to determine any discrepancies in the study’s outcomes.

While putting forward a potential procedural framework to assess the alignment between the policy vision of the Data Strategy and the practical implementation of existing and forthcoming regulations, it is important to note that the nature of our study precludes the incorporation of precise quantitative metrics. While the absence of quantitative measures is a limitation, it must be anticipated that our framework can serve as a valuable starting point, offering an alternative approach for future research endeavors. The authors encourage further exploration and refinement of this framework by researchers in the field, with the understanding that it may not be perfect but can be adapted and improved upon in subsequent studies. Ultimately, our goal is to contribute to the ongoing discourse surrounding data strategy evaluation and stimulate the development of more comprehensive assessment methodologies.

## 8   Conclusion

This study analysed the General Data Protection Regulation (GDPR), the Free-Flow of Non Personal Data Regulation (FFNPD), and the Open Data Directive (ODD) from the perspective of three different stakeholders, namely, individuals (consumers), businesses (insurance companies and car manufacturers), and public sector bodies.

The main aim of the study was to evaluate the effectiveness of the existing EU legal framework concerning access and reuse of data in comparison to what envisaged in the data strategy. To do so, the authors developed a framework to measure differences in access regimes across regulations and put it into practice on the case study of Event Data Recorders as example of privately held dataset.

Thanks to this methodology, the authors found that:

- from the perspective of individuals, in comparison to the GDPR (Articles 9,2 ; 15,1 ; 16 ; 17 ; 20), the combination of articles 4, 5, 6 and 26 of the Data Act give the user (individual and business) extended access and control to the EDR dataset by: (1) extending the CRUD actions to "direct access", "get availability"; (2) bolstering the CRUD actions with "immediate availability", or "including relevant data formats and data structures"; (3) extending the access to third-party businesses; (4) giving access to all dataset, (5) firstand thirdparties data; (6) and to vehicle data.
- from the perspective of businesses, the combinations of the provisions of the GDPR (Articles 6,1 ; 9,2) and the Articles 5, 7, 8, 13, 23, and 28 of the Data Act give the business) extend access and control to the EDR dataset by: (1) extending the CRUD actions to "direct access", "get availability"; (2) bolstering the CRUD actions with "immediate availability", or "including relevant data formats and data structures"; (3) extending the access to third-party businesses; (4) giving access to all dataset, (5) firstand thirdparties data; (6) and to vehicle data.
- from the perspective of public sector bodies, articles 14 and 15 of the Data Act give public bodies enlarged access to EDR data for the purposes of "tasks in the public interest" by: (1) extending the CRUD actions to "access", "use"; (2) bolstering the CRUD actions with "including metadata"; (3) extending access to the public sector; (4) giving access to non-personal data, (5) first parties data;

This analysis made it possible to observe that so far the GDPR is the regulation which offers the broadest possibility of accessing event data recorder data for the three stakeholders. Although the scope of the regulation is focused on personal data, it gives a broad range of tools for data access which was not provided by other regulations.

The proposed Data Act has the potential to create a conflict with the GDPR, particularly with regards to the distinction between personal and non-personal data. This is because the Data Act primarily concerns access to data generated by connected products, and does not differentiate between personal and non-personal data. Instead, it emphasizes technical aspects of data, such as format and quality, and includes the right to data portability for mixed datasets that contain both personal and non-personal data.

As a result of this study, the authors illustrate differences in EDR access rights across European Regulations. Although the Data Act has enhanced access and control over data held by businesses, the map highlights areas that require further development to enable practical data portability and data reuse rights. This challenge is compounded by the possibility of conflicts among overlapping Regulations concerning the same data, as well as uncertainties regarding future vertical or special regulations that will emerge with regards to vehicle data.

## Data Availability

The data for this article consists of bibliographic references, which are included in the References section. Zenodo: Blueprint: Data Access Enablers. From Stakeholders to Purpose, Through Use. https://doi.org/10.5281/zenodo.8347999 ^9^. This project contains the following extended data: blueprint.jpg. (A blueprint that can be used to trace Access to Data Rights). Zenodo: Stakeholders Access to Data - Standardized Schema. https://doi.org/10.5281/zenodo.8347919 ^8^. This project contains the following extended data: AccessRules.tex. (Schemato to streamline and enhance the understanding of access regimes while facilitating the creation of a machine-readable version for easier automation or embedding into software). Data are available under the terms of the Creative Commons Attribution 4.0 International license (CC-BY 4.0).
