# Supplementary material for: Measuring data access and re-use in the European Legal Framework for Data, from the General Data Protection Regulation (GDPR) law to the Proposed Data Act: the case of vehicle data
Source: Open Res Eur. 2023 Nov 7;3:192. [Version 1] doi: 10.12688/openreseurope.16468.1 (PMC10924165; doi:10.12688/openreseurope.16468.1)
Supplement: Peer review history

# Reviewer report: Reviewer response for version 1

**DOI:** 10.21956/openreseurope.17777.r38468

**Published:** 2024-03-08

**Authors:** Stergios Aidinlis

**Affiliations:** 1 Keele University, Keele, England, UK

This is a really interesting paper and I very much appreciate the opportunity to review it. The topic is very topical and fascinating and the authors are certainly very knowledgeable in the field. However, I think the article suffers from some critical weaknesses that prevent it from realising its potential to make a significant contribution to the literature. I note the following main points: 

 1. The false impression is given that some form of 'measurement' (see title) will happen to assess vehicle data access and re-use - this implies quantitative data on access to data for each of the stakeholders, something that is evidently not available right now, at least not presented in the present article. 

 2. Accepting that such measurement is not happening, we are then faced by the question of how methodologically sound the assessment conducted by the authors is. Our ability to assess this is perplexed by the fact that the authors draw on the CRUD framework in the introduction, but then do not elaborate enough on the implementation of this framework when they actually compare different legislative avenues for stakeholders (in sections 5 and 6) - CRUD is only mentioned again on page 17 and then in the conclusion (in section 8), but the reader struggles to understand how the findings resulted from the preceding legal analysis. We needed clear reference to CRUD throughout sections 5 and 6 to understand the points that the authors are trying to make. 

 3. Speaking of the legal analysis, and more specifically sections 5 and 6, I feel that, unfortunately, the tripartite distinction between individuals, businesses and public bodies does not work as the authors intended it to work. It results in significant repetitions, as a lot of provisions are applicable in an almost identical manner and the text repeats points that were made before. For example, the beginning of section 6 elaborates on the notion of European Common Data Spaces, something that is repeated in the beginning of sub-section 6.4. Almost identical discussions of article 3 of the Data Act are provided under sub-sections 6.1 and 6.2. I would opt for a more thematic treatment of issues under the data act, only drawing distinctions between the stakeholders when this is necessary to illuminate the analysis. 

 4. The paper lacks a clear articulation of the application of the concept of 'personal data' in data protection legislation (mainly the GDPR) to the context of vehicle data and how it overlaps with the definition of 'particular data' espoused by the authors. There are some tangential references to this issue (e.g., the reference to the *Nowak *case), but it is a fundamental issue that underpins the whole of the analysis in section 5, and would benefit from a comprehensive analysis under section 4.1. 

 5. The main Data Act innovation in terms of public sector access to privately held data, i.e., article 15, is only mentioned in passing and not thoroughly analysed in sub-section 6.7. 

 There are other minor things e.g., it would be good to have some more articulation of the normative underpinnings of the entitlements of the rights of different stakeholders to vehicle data (M. Mazzucato's work could be instructive here), or it would be good to reflect more on the application of the right to erasure to vehicle data (how far back should insurance companies be entitled to go to assess our qualities as drivers? Is my driving capability 20 years before relevant under the law?) - but these are secondary points. 

If applicable, is the statistical analysis and its interpretation appropriate?

I cannot comment. A qualified statistician is required.

Is the study design appropriate and is the work technically sound?

Partly

Is the work clearly and accurately presented and does it engage with the current literature?

Partly

Are the conclusions drawn adequately supported by the results?

Partly

Are sufficient details of methods and analysis provided to allow replication by others?

Not applicable

Are all the source data and materials underlying the results available?

Yes

Reviewer Expertise:

data protection law; AI; data sharing

I confirm that I have read this submission and believe that I have an appropriate level of expertise to confirm that it is of an acceptable scientific standard, however I have significant reservations, as outlined above.

# Reviewer report: Reviewer response for version 1

**DOI:** 10.21956/openreseurope.17777.r38462

**Published:** 2024-03-08

**Authors:** Dr. Md. Toriqul Islam

**Affiliations:** 1 Department of Law, Independent University, Bangladesh (IUB), Dhaka, Bangladesh

The paper, **“Measuring Data Access and Reuse in the European Legal Framework for Data: From the General Data Protection Regulation (GDPR) Law to the Proposed Data Act: The Case of Vehicle Data”** by Crepax *et al.*, analyses legal and technical issues surrounding acquiring, sharing, and repurposing vehicle data, particularly from black boxes and event data recorders. The authors propose a methodology to evaluate data access and reuse levels under different regulations, applying it to vehicle data within the existing and proposed EU legal frameworks.

**Strengths:**

- The paper addresses a timely and relevant topic, as vehicle data is valuable for various stakeholders, such as consumers, insurers, car makers, public authorities, and service providers.
- It offers valuable insights into the complexities and challenges of measuring data access and reuse within the European context, contributing to the existing body of knowledge on the subject.
- The paper effectively communicates its objective of measuring data access and reuse, providing clarity to the reader about the research goals.
- The methodology to measure data access and reuse using categories of data, data use purposes, and involved actors is original and innovative.

**Weaknesses:**

- The title of the paper could be more concise and impactful, as it includes additional prepositions and unnecessary details about the GDPR.
- While the paper starts strong in terms of language, its quality declines throughout. This is particularly evident in the sentence structure, which relies on long and complex sentences, and the inconsistent use of both British and American English. Additionally, the referencing style lacks consistency, with inconsistencies found in either footnotes or in-text citations. To ensure textual accuracy, the authors are encouraged to use a professional proofreading service.
- The paper does not discuss sufficiently the ethical and social implications of data access and re-use, such as the potential risks of discrimination, manipulation, or harm to individuals or groups, or the need for transparency, accountability, and consent mechanisms.
- The paper does not consider the possible alternatives or complements to the proposed Data Act, such as self-regulation, contractual agreements, technical standards, or the role of other actors, such as data intermediaries, data trusts, or data cooperatives.

**Areas for improvement:**

- The paper could benefit from providing more empirical evidence or data to support its claims and assumptions, such as case studies, surveys, interviews, or experiments, or from acknowledging the gaps and limitations of the existing data sources.
- The paper could improve its analysis by incorporating the ethical and social dimensions of data access and reuse and by discussing the possible trade-offs, conflicts, or synergies between different values, interests, and rights.
- The paper could broaden its perspective by exploring the possible alternatives or complements to the proposed Data Act and by examining the role and impact of other actors and mechanisms in the data ecosystem.
- The paper could enhance its methodology by addressing its limitations or challenges and by providing more details, examples, or illustrations of how it works and how it can be applied to other cases or contexts.
- The paper could include a comparative analysis of data access and reuse regulations of some other jurisdictions than the EU.
- The paper could benefit from a comparative analysis of data access and reuse regulations in other jurisdictions, providing a broader perspective on the topic.
- Some sections of the paper could be further clarified to improve readability and comprehension, particularly regarding technical or legal terminology.

**Recommendation:**

 The title of the paper is grammatically correct and conveys the paper's topic clearly. However, it could be improved for conciseness and impact in an academic setting. The authors can consider removing unnecessary words like "the" before "General Data Protection Regulation" and "the" before "Proposed Data Act". Accordingly, the revised title can be as follows:

**Measuring Data Access and Reuse in the European Legal Framework: From GDPR to the Proposed Data Act.**

 Based on the above review, I would recommend the paper to be **approved with reservations**, as it has some strengths and original contributions but also some weaknesses and areas for improvement. The paper could be **accepted for indexing if the authors revise and improve it **according to the suggestions and comments provided.

 The authors should also carefully review the attached PDF linked here for any necessary corrections to their paper.

If applicable, is the statistical analysis and its interpretation appropriate?

Not applicable

Is the study design appropriate and is the work technically sound?

Yes

Is the work clearly and accurately presented and does it engage with the current literature?

Yes

Are the conclusions drawn adequately supported by the results?

Yes

Are sufficient details of methods and analysis provided to allow replication by others?

Yes

Are all the source data and materials underlying the results available?

Yes

Reviewer Expertise:

Data Protection Law, Privacy Law, Cybersecurity Law, Law and Technology

I confirm that I have read this submission and believe that I have an appropriate level of expertise to confirm that it is of an acceptable scientific standard, however I have significant reservations, as outlined above.

# Reviewer report: Reviewer response for version 1

**DOI:** 10.21956/openreseurope.17777.r36402

**Published:** 2024-02-22

**Authors:** chiara Gallese Nobile

**Affiliations:** 1 School of Engineering, Universita Carlo Cattaneo (Ringgold ID: 18963), Castellanza, Lombardy, Italy; 2 Department of Electrical Engineering, Eindhoven University of Technology, Eindhoven, Netherlands Antilles; 3 ISLC - Information Society Law Center, Universita degli Studi di Milano (Ringgold ID: 9304), Milan, Lombardy, Italy; 4 Law Department, Universita degli Studi di Torino (Ringgold ID: 9314), Turin, Piedmont, Italy

This article explores the issues related to vehicle data use for different stakeholders and the challenges regarding data portability in light of the EU Digital Strategy. The authors explores the challenged posed by the General Data Protection Regulation (GDPR), the Free-Flow of Non Personal Data Regulation (FFNPD), and the Open Data Directive (ODD).

 The article is well-written and interesting. It tackles some issues that legal scholarship has yet to explore in depth, and it is related to a field that would require more attention from legal scholars. The quality of the article is very good. I appreciate the opportunity to review the manuscript.

 I have identified a few areas that require attention and improvement. Addressing these points will significantly improve the paper and contribute to advancing the field.

 1. From a methodological point of view, the article would greatly benefit from contextualizing the discussion in light of the existing literature. The article's findings should be discussed by confronting the relevant works in the field, commenting and citing different opinions of colleagues (those who agree and those who do not agree with the conclusions). A significant number of relevant articles for the discussion should be cited unless strict page constraints mandate a succinct bibliography (e.g., what happens in conference proceedings). This paper would significantly improve if every paragraph carried several references to justify each claim made by the authors. Each claim should be referred to a relevant source. For example, several scholars have commented on the Google Spain case, but no one is cited. Other scholars have already said most of the content described in paragraphs 6.1, 6.2, and 6.3. Paragraph 6.5 is also mostly repeating what has already been noted by other scholars. I listed below some examples of relevant citations on the Data Act, but the authors can find more references in the most influential law journals. 

 2. The decision to exclude part of the relevant laws on the topic, such as the Data Governance Act, should be justified by methodological reasons, not by "practical reasons". The article has enough "redundant content" to cut to include the missing regulations, if necessary, and improve readability. 

 3.The article would benefit from restructuring the content and topics addressed, as some sections are very long and could be integrated differently with the rest of the narrative (e.g., the stakeholders). Part 6 could be completely omitted without compromising the value of the paper.

 Some parts of the article are overly generic, redundant, or already repeated in many other articles. I advise cutting unnecessary content or rewriting fluffy paragraphs such as "In summary, the use of iBBs for car insurance purposes presents a balancing act between the potential benefits of lower premiums for consumers and the potential loss of autonomy. It is important to adhere to data protection principles while ensuring that the legitimate interests of both parties are maintained."

 4. The scope of the article seems too broad since it has an ambitious goal of presenting the point of view of multiple stakeholders who have different needs and are subjected to different regulations. Restricting the scope to only one of those stakeholders (e.g., public entities, researchers, or insurance companies) would improve the article's focus.

 5. Some other comments could be made on the text, such as clarifying some concepts (e.g., explaining at the beginning what types of data are generated, explaining the concept of data quality in more detail, and explaining the consequences of selective data portability). For example, the article would improve if some assumptions and implications were made explicit: when the authors say, "if an individual chooses

 to port only their driving performance data but not data

 related to alcohol detection, it could create an imbalanced picture of their overall risk profile as a driver, resulting in subjective and distorted reality and reintroducing information asymmetries that data recorders are supposed to reduce.

 Similarly, there may be situations where an individual wants to sell a car embedded with mEDR data related to malfunctions, but only selectively ports certain data, potentially compromising the safety of the next owner. Thus, it is necessary to find a solution that balances the individual’s right to informational self-determination with considerations of good faith and fairness towards the recipient of the ported data, as well as public safety." they do not mention that there are already contractual and criminal laws to sanction such behaviors. Since data portability is an important right introduced by GDPR and the Data Act, what are the authors suggesting? In their opinion, how should the law change to address these issues?

 Overall, I think the article is very good and can be easily improved to reach its full potential.

If applicable, is the statistical analysis and its interpretation appropriate?

Partly

Is the study design appropriate and is the work technically sound?

Yes

Is the work clearly and accurately presented and does it engage with the current literature?

Partly

Are the conclusions drawn adequately supported by the results?

Yes

Are sufficient details of methods and analysis provided to allow replication by others?

Not applicable

Are all the source data and materials underlying the results available?

Yes

Reviewer Expertise:

Data Act, Data Protection Law, Data Ethics, Reuse of Data, GDPR, AI and Law

I confirm that I have read this submission and believe that I have an appropriate level of expertise to confirm that it is of an acceptable scientific standard, however I have significant reservations, as outlined above.

## References

[1] : Governance of IoT Data: Why the EU Data Act Will not Fulfill Its Objectives. *GRUR International* .2023;72(2) : 10.1093/grurint/ikac107 120-135 10.1093/grurint/ikac107

[2] : The Data Act Proposal and the Problem of Access to In-Vehicle Data and Resources. *SSRN Electronic Journal* .2022; 10.2139/ssrn.4115443 10.2139/ssrn.4115443

[3] : European Union ∙ The Data Act: The Next Step in Moving Forward to a European Data Space. *European Data Protection Law Review* .2022;8(1) : 10.21552/edpl/2022/1/16 108-114 10.21552/edpl/2022/1/16

[4] : The EU Data Act in Context: A Legal Assessment. *SSRN Electronic Journal* .2023; 10.2139/ssrn.4584781 10.2139/ssrn.4584781

[5] : Shaping Markets: A Critical Evaluation of the Draft Data Act. *SSRN Electronic Journal* .2022; 10.2139/ssrn.4222376 10.2139/ssrn.4222376

[6] : Caught in the Acts: Framing Mandatory Data Access Transactions under the Data Act, Further EU Digital Regulation Acts, and Competition Law. *Journal of European Competition Law & Practice* .2023;14(2) : 10.1093/jeclap/lpac059 67-82 10.1093/jeclap/lpac059

[7] : THE EU DATA ACT. *CEPS* .2022;

[8] : the data act proposal. *irdg* .2023;

[9] : A first commentary to the proposal for a new Regulation on fair access and use of data (Data Act).2022;

[10] : Smart Working e sistemi di controllo dei risultati e dell'attività.2019;

# Reviewer report: Reviewer response for version 1

**DOI:** 10.21956/openreseurope.17777.r38035

**Published:** 2024-02-20

**Authors:** Marco De Vincenzi

**Affiliations:** 1 Italian National Research Council, Rome, Italy

The article explores challenges and opportunities related to accessing and repurposing vehicle data from black boxes and event data recorders. It discusses potential benefits for consumers, competitors, and public institutions, such as data portability and intelligent mobility solutions. It addresses a relevant topic in a systematic and consistent manner, providing interesting comparisons and points of view for future data management in the automotive industry. 

 Minor Comments:

 - Section 2.2: "Structure of the Paper" - The description of Section 4 should be condensed and moved to another section.

 - Section 2.2: "Structure of the Paper" - The last part starting from "In this paper..." seems more like a contribution. I suggest splitting the section into structure and contributions.

 - UN Regulation No. 160, which applies to the approval of vehicles of categories M1 and N1, should be explicitly mentioned in the paper.

 Typos:

 - General Data Protection Regulation (GDPR) - Use the acronym after the first citation.

 - Section 4.1: "Particular Data and Aggregated Data" - "Namely,..." Why is "Particular" capitalized?

 - Section 4.3.3: "As Abdulhafedh points out,...": Add reference.

 - Section 4.3.3: "As Hampton et al., points out,...": Add reference.

 - Section 5.1.2: "and emergency responders [?]": Typo.

 - The link "https://doi.org/10.5281/zenodo.83479999" does not work.

 Suggestions:

 - Compare regulations outside Europe, such as the California Consumer Privacy Act or the Chinese Provisional Regulations on Data Security Management in the Automotive Industry. It could be useful to have a short paragraph or a section that considers the comparison with non-EU regulations.

If applicable, is the statistical analysis and its interpretation appropriate?

Not applicable

Is the study design appropriate and is the work technically sound?

Yes

Is the work clearly and accurately presented and does it engage with the current literature?

Yes

Are the conclusions drawn adequately supported by the results?

Yes

Are sufficient details of methods and analysis provided to allow replication by others?

Yes

Are all the source data and materials underlying the results available?

Yes

Reviewer Expertise:

Automotive Cybersecurity and privacy

I confirm that I have read this submission and believe that I have an appropriate level of expertise to confirm that it is of an acceptable scientific standard.
